# Three-Dimensional Hierarchical Nanostructured Cu/Ni–Co Coating Electrode for Hydrogen Evolution Reaction in Alkaline Media

**DOI:** 10.1007/s40820-015-0049-1

**Published:** 2015-06-30

**Authors:** Ning Wang, Tao Hang, Dewei Chu, Ming Li

**Affiliations:** 1 State Key Laboratory of Metal Matrix Composites, School of Material Science and Engineering, Key Laboratory for Thin Film and Microfabrication of Ministry of Education, Shanghai Jiao Tong University, Shanghai, 200240 People’s Republic of China; 2 School of Materials Science & Engineering, University of New South Wales, Sydney, NSW 2052 Australia

**Keywords:** Electrodeposition, Ni–Co alloy, Hierarchical, Hydrogen evolution reaction

## Abstract

In this work, three-dimensional hierarchical nickel–cobalt alloy coating for hydrogen evolution cathode was fabricated by electrodeposition processes. The coatings’ morphology evolves from sea cucumber-like nanostructure to caterpillar-like one with the increase of cobalt content. A large amount of nanometric “steps,” served as the active sites for hydrogen evolution reaction, were observed. According to Tafel polarization measurements, the exchange current density of the as-synthesized coating with hierarchical nanostructure was 21.9 times compared with that of flat nickel coating. In addition, the hierarchical coating also displayed good electrochemical stability from the galvanostatic test.

## Introduction

The electrochemical production of hydrogen by alkaline water electrolysis is one of the most promising methods for renewable energy sources [1, 2]. Due to the high energy consumption of the water electrolysis, considerable efforts have been made in recent years to explore new cathodic materials that can minimize hydrogen over-potential and promote hydrogen evolution. Generally, noble metals are the most active materials for hydrogen evolution reaction (HER), although they are very expensive [3, 4]. The selection criterions of the alternative electrode for water electrolysis include low hydrogen over-potential, high electrocatalytic activity, high electrical conductivity, electrochemical stability, and reasonable cost. Alloying is a common approach to improve the intrinsic activity of hydrogen evolution of metal electrodes [5]. In particular, nickel and its alloys are well-known non-precious metal electrocatalysts for hydrogen production in alkaline electrolytes because of their special corrosion resistance and highly intrinsic electrocatalytic activity toward HER. Meanwhile, increasing the surface area of the electrodes is also recognized as a way to reduce undesirable polarization loss. Several electrodes with excessive surface area have been reported in recent years, such as the commercial Raney type electrodes [3–5], anodic aluminum oxide-assisted electrodeposited Ni nanowires [4], SnO_2_ hierarchical architectures [5], double-template electrodeposited three-dimensional porous nickel [6], and so on.

On the other hand, nanostructured materials have attracted considerable attention recently for their prospects in magnetic [7–9], electronic [10], and electrochemical [11] devices applications. Especially for one-dimensional nanomaterial, such as nanocone and nanowire, electrons are free only in one direction and confined in the other directions, which is a driving force that provides the electrons faster allowing them to interface for rapid reactions and high electroactivity [12]. The larger specific surface area and the facile vectorial electron transport along the axis of one-dimensional nanostructured materials make them a promising candidate for catalytic materials.

The metallic nanocones arrays fabricated by the template-free electrodeposition method have previously been reported for their enhanced magnetic properties and application in lithium battery current collectors, yet there are no scientific reports available regarding their application for HER catalyst [13]. In this work, a three-dimensional hierarchical nanostructured Cu/Ni–Co coating was fabricated by electroplating Ni–Co alloy nanocones array onto Cu microcone array, varying the Co composition from 0 to 30 % in the alloy. This unique hierarchical nanostructured Cu/Ni–Co coating with larger specific surface area, which can provide more electrochemical active sites, is demonstrated to have enhanced HER performance.

## Experimental

### Electrode Preparation

The Ni–Co alloy nanocone arrays were electrodeposited onto the Cu microcone arrays. The Cu microcones were obtained by the electroless deposition method [14]. Subsequently, Ni–Co alloy nanocone arrays were electroplated on the as-prepared Cu microcones arrayed substrate at a constant current of 0.01 A cm^−2^ and 60 °C for 5–10 min. The content of NiCl_2_·6H_2_O in the plating bath was fixed at 1 mol L^−1^, while the content of CoCl_2_·6H_2_O was varied in the range of 0–0.05 mol L^−1^. H_3_BO_3_ (0.5 mol L^−1^) and ethylene diamine dihydrochloride (1.0 mol L^−1^) were added as buffer agent and crystallization modifier, respectively. The pH value of the plating bath was adjusted to 4.0 using ammonium hydroxide. All the chemicals purchased from Sinopharm Chemical Reagent Co. were of analytical grade and used without any further purification. In addition, flat Ni plate was also evaluated for comparison.

### Characterization

The morphologies of the hierarchical structured Cu/Ni–Co were characterized by field emitting scan electronic microscope (FE-SEM, FEI SIRION200) and transmission electron microscope (TEM, JEM 2100F). The elemental composition of the coating was investigated by energy dispersive X-ray detector (EDX, INCA OXFORD). The surface chemical composition was investigated by X-ray photoelectron spectroscopy (XPS, Kratos AXIS Ultra^DLD^) using a monochromatic Al Kα source (1486.6 eV).

HER on the electrodes was investigated by means of Tafel curves and Galvanostatic curves measured in 1 mol L^−1^ NaOH solution using an electrochemical workstation (CHI660, Shanghai Chenhua Instrument Co. Ltd.) at room temperature. A standard three-electrode system was used with a 1 mol L^−1^ NaOH Hg/HgO electrode as the reference electrode and a Pt plate as the counter electrode. Galvanostatic responses were recorded at a current density of 0.2 A cm^−2^ for 24 h.

## Results and Discussion

**Fig. 1:**
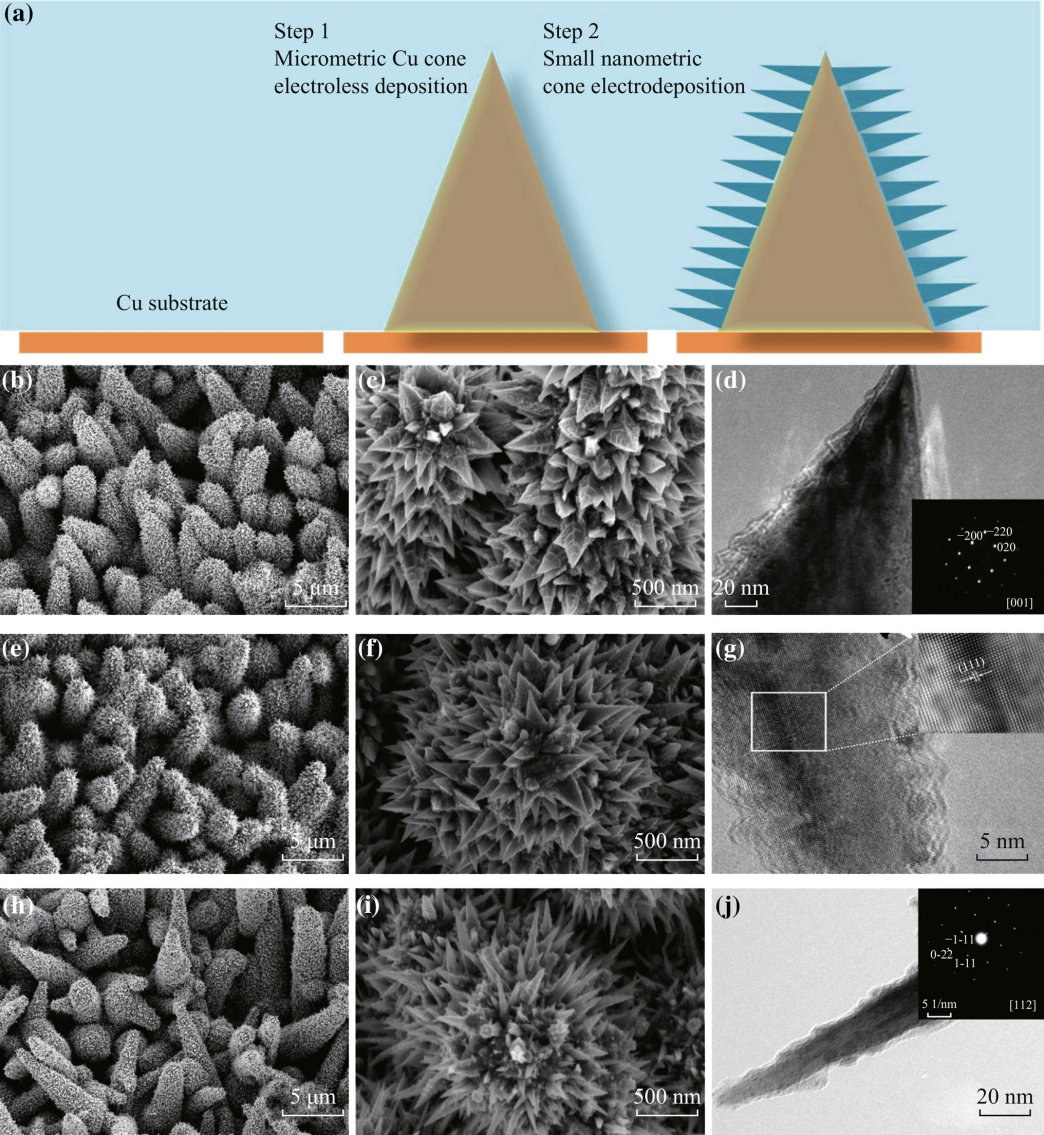
**a** The formation schematic of the hierarchical structured electrodes. SEM images of varying hierarchical structures with different magnification: **b**, **c** Cu/Ni, **e**, **f** Cu/Ni–Co 10, **h**, **i** Cu/Ni–Co 30. TEM images of the single nanocone **d** Ni, **j** Ni–Co 30, and the *inset* is the corresponding SAED image. **g** HRTEM image of Ni–Co 10 nanocone, and the *inset* is the enlarged view of the *white encircled* part

The Ni–Co alloy nanocones arrays were electroplated onto Cu microcone arrays with varying molar ratio of Co^2+^ and Ni^2+^ in the plating bath. The electrolyte composition and the alloy composition of the alloy measured by EDS are shown in Table 1. According to the atomic percentage of cobalt in alloy, as-prepared alloy coatings are denoted Ni, Ni–Co 10, and Ni–Co 30, respectively, and the corresponding hierarchical coatings are denoted Cu/Ni, Cu/Ni–Co 10, and Cu/Ni–Co 30, respectively. The content of cobalt was found to be much higher than that in the plating bath. However, it is hardly to find any persuasive explanation for this anomaly, and it was classified as an anomalous codeposition [15–20]. Figure 1a shows the formation schematic of the hierarchical nanostructures. First, Cu microcones were electroless plated on the copper substrate. Subsequently, the substrate with as-prepared Cu microcones was immersed into the plating bath to form Ni–Co alloy nanocone coating on the surface of the Cu microcones.

**Table 1:** Electrolyte and alloy composition of different samples and the corresponding electrochemical parameters

Coating name	Electrolyte composition		Alloy composition		Bc (mV dec^−1^)	*i*_0_ (A cm^−2^)	log*i*_200_ (A cm^−2^)
	Ni^2+^ (mol L^−1^)	Co^2+ ^(mol L^−1^)	Ni (at %)	Co (at %)			
Cu/Ni	1	–	100	–	121.3	1.4 × 10^−5^	−3.98
Cu/Ni–Co 10	1	0.025	90.65	9.35	124.2	2.5 × 10^−5^	−3.92
Cu/Ni–Co 30	1	0.05	73.07	26.93	128.6	4.6 × 10^−5^	−3.58
Flat Ni plate	–	–	–	–	123.4	2.1 × 10^−6^	−4.47

Figure 1b–c, e–f, h–i shows the SEM images of the hierarchical nanostructured Cu/Ni–Co coating. Two layers were observed. The first layer is micrometric protrusions (Cu cones), and the second layer is nanometric cones. The average height of Cu cones is about 7–8 μm, and the root diameter is about 1–2 μm. Pure Ni nanocone was about 500 nm in height and 250 nm in root diameter. With the increase of cobalt content in alloy, the nanocones become sparser and slenderer, i.e., the aspect ratio of the nanocones is increased. This may be attributed to the chemical potential difference of the solid–liquid interface and the synergistic effect of twinning [21]. Consequently, the morphology of the hierarchical structure changes from sea cucumber-like to caterpillar-like correspondingly. The TEM images and the high-resolution TEM (HRTEM) image of Ni–Co alloy nanocones are also shown in Fig. 1. Many sharp edges were observed on the surface of the nanocone (shown in Fig. 1d, g, j), which are actually the steps of the screw dislocation. According to our previous report, the growth mechanism of these electrodeposited nanocones can be explained by the screw dislocation growth on a real crystal surface with defects, and the surface was confined by the (100) and (111) side planes [21, 22]. However, the pure Ni nanocone looks thick, and the crystal growth orientation is close to (110) (Fig. 1d and the inset). With the increase of Co content, the sharpness of the nanocone is greatly increased while growth orientation remained unchanged (Fig. 1j and the inset). The HRTEM image of the Ni–Co 10 shows perfect lattice fringes with an inter-planar distance of 0.203 nm (Fig. 1g and the enlarged view of the inset figure), corresponding to the (111) plane of face-centered cubic nickel. It indicates that Co and Ni formed nickel-based solid solution nanocones.

**Fig. 2:**
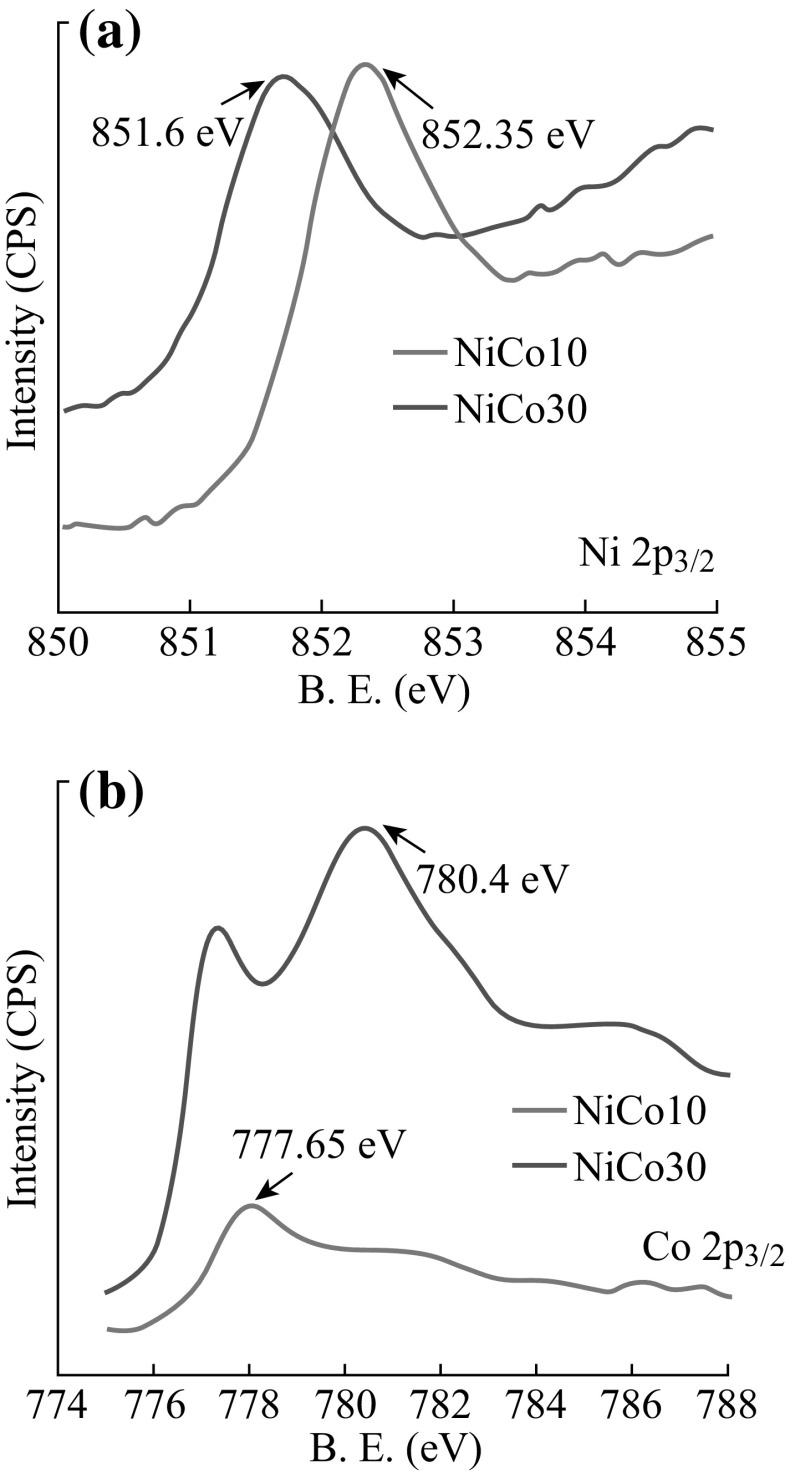
XPS spectra taken from the nanostructured electrode of Ni–Co 10 and Ni–Co 30: **a** Ni 2p_3/2_, **b** Co 2p_3/2_

In order to further confirm the formation of Ni–Co alloy nanocones, both Cu/Ni–Co 10 and Cu/Ni–Co 30 were analyzed by XPS. Figure 2a, b displays the peaks of Ni 2p_3/2_ and Co 2p_3/2_. Obvious peaks at 852.4 and 851.6 eV can be attributed to the binding energy of Ni 2p_3/2_, whereas for Co, the peaks located at 777.7 and 780.4 eV can be assigned to the binding energies of Co 2p_3/2_. Because of alloying, the signals of metallic bonding energies in Ni–Co alloys are slightly shifted compared with the pure nickel and cobalt bulk metals. Both of them reveal the formation of Ni–Co alloy nanocones.

**Fig. 3:**
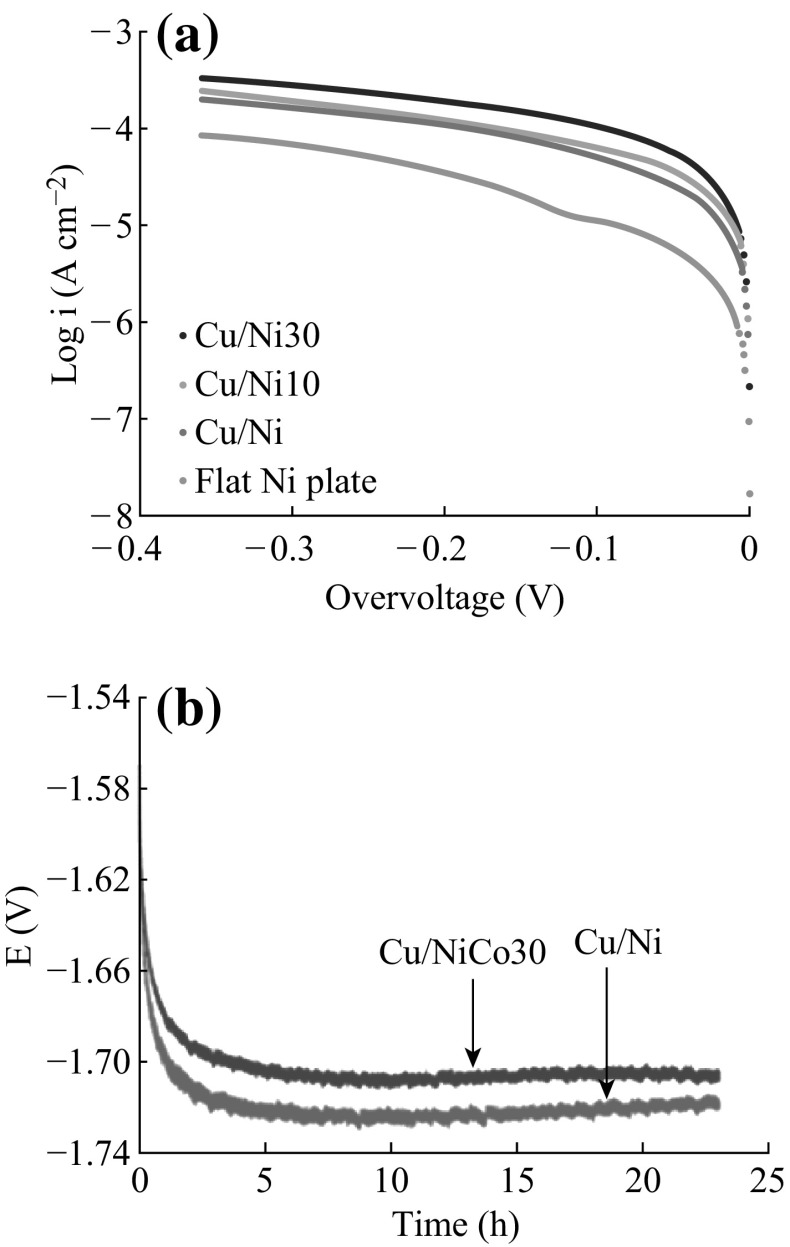
**a** The cathodic current–potential curves in HER for the three hierarchical structures and the flat Ni. **b** The cathodic potential curves in HER for Cu/Ni–Co 30 and Cu/Ni coatings

Figure 3a shows the cathodic Tafel curves of the hierarchical nanostructured electrodes measured in 1 mol L^−1^ NaOH solution. Some critical electrochemical kinetic parameters calculated from the Tafel curves, such as the Tafel slope, the value of exchange current density (*i*_0_), and the current density at the overvoltage of 200 mV (*i*_200_), are listed in Table 1. The values of both *i*_0_ and *i*_200_ measured on four different electrodes have the same trend: Cu/Ni–Co 30 > Cu/Ni–Co 10 > Cu/Ni > flat Ni plate.

The exchange current density *i*_0_ of the Cu/Ni nanostructured electrode is 1.4 × 10^−5^ A cm^−2^, which is about six times as high as that of the flat Ni plate. The enhanced *i*_0_ of the Cu/Ni nanostructured electrode can be mainly ascribed to the excessive specific surface area of the hierarchical structures and the facile vectorial electron transport along with a great amount of nanometric “steps,” which is derived from the process of crystal growth. Usually, the formation of hydrogen bubbles at the surface will lead to an increased resistance due to the decrease in available active sites for HER. However, the hydrogen bubbles can easily detach from the surface with sharp edges [2, 23]. The hierarchical nanostructured coating, with its high population of “steps,” has a structural advantage which can decrease the resistance, thereby leading to an enhancement of the catalytic activity. Besides, the unique micro-nano “open” structure is conducive to the discharge of H_2_ bubbles [3]. Electrolyte transported well into the bottom of the nanostructure to make good contact with electrodes. Thus, the effective current density was increased.

With the increase of Co content in the alloy, the exchange current density of the hierarchical nanostructured electrode is up to over 20 times as high as that of the flat Ni plate electrode. It indicates that not only the excessive surface area but also the synergistic effect of nickel and cobalt plays an important role in the enhancement of HER. The similar effect for the other alloy coatings of transition metals, such as NiMo, NiW, NiFe, and NiCu, was also reported in literature [24, 25].

Besides the HER, the galvanostatic behaviors of these nanostructured electrodes were also measured for lifetime determination. Figure 3b shows the representative voltage profiles for the Cu/Ni and Cu/Ni–Co 30 nanostructured coatings. As the reaction proceeding, the voltage profiles of both coatings stabilized after 5-h measurement, and the Cu/Ni–Co 30 coating shows a relatively positive operating cathodic potential with lower energy consumption during the whole process, which indicates that the electrode structures are not damaged after a long time release of gas bubbles.

## Conclusions

In summary, Ni–Co alloy-based three-dimensional hierarchical nanostructures with sharp edges were prepared by a simple two-step electroless and electro-deposition approach, which presents excellent catalytic performances of the HER. The enhanced exchange current density of the hierarchical nanostructures can be mainly ascribed to the increase of specific surface area, the nanometric “steps”, and the synergistic effect of nickel and cobalt atoms. These hierarchical nanostructured coatings demonstrated good durability in galvanostatic lifetime determinations.
